# A metabonomic study to explore potential markers of asymptomatic hyperuricemia and acute gouty arthritis

**DOI:** 10.1186/s13018-023-03585-z

**Published:** 2023-02-13

**Authors:** Wei Wang, Jun Kou, Mingmei Zhang, Tao Wang, Wei Li, Yamen Wang, Qingyun Xie, Meng Wei

**Affiliations:** 1 Department of Orthopedics, General Hospital of Western Theater Command, Rongdu Avenue No. 270, Chengdu, 610000 People’s Republic of China; 2 College of Medicine, Southwest Jiaotong University, North Section 1 No. 111, Second Ring Road, Chengdu, 610000 People’s Republic of China; 3 Department of Rheumatism and Immunology, The General Hospital of Western Theater Command, Tianhui Road 270, Chengdu, 610000 People’s Republic of China

**Keywords:** Metabonomics, Acute gouty arthritis, Asymptomatic hyperuricemia, Biomarkers, Diagnosis

## Abstract

**Background:**

Acute gouty arthritis (AGA) is a metabolic disease with acute arthritis as its main manifestation. However, the pathogenesis of asymptomatic hyperuricemia (HUA) to AGA is still unclear, and metabolic markers are needed to early predict and diagnose. In this study, gas chromatography (GC)/liquid chromatography (LC)–mass spectrometry (MS) was used to reveal the changes of serum metabolites from healthy people to HUA and then to AGA, and to find the pathophysiological mechanism and biological markers.

**Methods:**

Fifty samples were included in AGA, HUA, and healthy control group, respectively. The metabolites in serum samples were detected by GC/LC–MS. According to the statistics of pairwise grouping, the statistically significant differential metabolites were obtained by the combination of multidimensional analysis and one-dimensional analysis. Search the selected metabolites in KEGG database, determine the involved metabolic pathways, and draw the metabolic pathway map in combination with relevant literature.

**Results:**

Using metabonomics technology, 23 different serum metabolic markers related to AGA and HUA were found, mainly related to uric acid metabolism and inflammatory response caused by HUA/AGA. Three of them are completely different from the previous gout studies, nine metabolites with different trends from conventional inflammation.

**Conclusions:**

In conclusion, we analyzed 150 serum samples from AGA, HUA, and healthy control group by GC/LC–MS to explore the changes of these differential metabolites and metabolic pathways, suggesting that the disease progression may involve the changes of biomarkers, which may provide a basis for disease risk prediction and early diagnosis.

## Background

Gout is an inflammation and tissue damage caused by monosodium urate (MSU) deposition in bone joints, kidneys, and subcutaneous areas. It is directly related to hyperuricemia caused by purine metabolism disorder and/or reduction of uric acid excretion [1]. With the change of lifestyle and diet structure, the prevalence rate is increasing year by year, and the prevalence rate in Pacific countries is the highest [2]. Gout includes acute gouty arthritis (AGA) and chronic gouty arthritis (CGA). The accumulation of excessive uric acid crystals in tissues and blood causes AGA, which is manifested as white or yellow MSU crystals deposited in soft tissues microscopically or macroscopically. The main symptoms of AGA are joint redness and swelling, accompanied by severe pain. Asymptomatic hyperuricemia (HUA) refers to that under the normal purine diet, the blood uric acid of fasting men and postmenopausal women is > 420 µmol/L (7.0 mg/dL) and that of women is > 360 µmol/L (6.0 mg/dL). The uric acid level of HUA is mostly higher than the normal value according to the physical examination, and the patients generally have no obvious symptoms [3]. HUA is a prerequisite for the development of AGA. When the serum dissolved uric acid level is 6.8 mg/dL higher than the physiological saturation threshold, sodium urate is easier to crystallize, and the risk of developing AGA increases [4]. The typical development of gout can start from HUA without MSU crystal deposition, to MSU crystal deposition in asymptomatic gout, to AGA, and finally to gout stone and CGA [5]. However, studies have shown that up to 76% of HUA patients have no crystal deposition, and 10% of HUA patients will not have gout for life [6]. This makes us realize that the relationship between HUA and gout may not be a simple causal relationship. And most people seldom check their uric acid level. They often find out that they have high uric acid or gout after falling ill. Therefore, it is particularly important to explore the pathogenesis of HUA and AGA and find some other biomarkers to assist in the diagnosis.

Metabonomics is a biological technology used to identify the global metabolic spectrum of endogenous small molecular substances. It can also be used to characterize the different physiological and pathological characteristics of organisms under external physical, chemical, and environmental stimuli. In recent years, metabonomics has been widely used in disease diagnosis and detection, which is of great significance to understand the pathogenesis of disease [7]. Liquid chromatography (LC)–mass spectrometry (MS) and gas chromatography (GC)–MS are the most commonly used mass spectrometry platforms in metabonomics. GC–MS has greater advantages in distinguishing volatile compounds and low molecular weight substances, while LC–MS usually analyzes thermally unstable, non-volatile, and polar compounds. The combination of GC–MS and LC–MS can increase metabolome coverage or validation [8].

In this study, GC/LC–MS was used to comprehensively analyze the serum metabolites of normal people, HUA and AGA patients, and the changes of metabolites in three groups of patients were screened out. Taking these differential metabolites as markers for early diagnosis of HUA and AGA will help to prevent the occurrence and progress of HUA and AGA, and reduce the burden on individuals, families, and society. To the best of our knowledge, this is the first metabonomic study of the serum of HUA and AGA using GC/LC–MS.

## Methods

### Participants

This study selected participants who went to the outpatient and inpatient clinics of the General Hospital of Western Theater Command from August 2019 to June 2020. According to the diagnostic criteria of HUA and AGA, the inclusion and exclusion criteria were set. Fifty patients of HUA and 50 patients of AGA were selected. Fifty healthy volunteers were recruited from the healthy people who went to the physical examination center for routine physical examination as the control group. The participants in the three groups were left with fasting blood from the morning. Inclusion criteria of HUA group: (1) HUA was defined as blood uric acid > 420 µmol/L (7.0 mg/dL) in fasting men and postmenopausal women and > 360 µmol/L (6.0 mg/dL) in women under normal purine diet, (2) first discovery and diagnosis, (3) age ≥ 18 years old, ≤ 80 years old, regardless of gender, (4) the patient signed the informed consent form. The inclusion criteria of AGA group were as follows: (1) the diagnostic criteria of AGA by American Society of Rheumatology in 1997 were adopted, (2) patients who have been found and diagnosed for the first time without medical treatment, (3) age ≥ 18 years old, ≤ 80 years old, regardless of gender, (4) the patient signed the informed consent form. The inclusion criteria of the healthy control group (HCG): (1) age ≥ 18 years old, ≤ 80 years old, regardless of gender, (2) not diagnosed as high urea and AGA, (3) the patient signed the informed consent form. The exclusion criteria were applicable to three groups: (1) drug treatment; (2) women during menstruation, pregnancy, lactation, and psychosis; (3) those who have participated in clinical trials of other drugs within three months, (4) who have other diseases that may cause metabolic abnormalities or have addictive tendencies. This study requires that all volunteers review and sign a form of informed consent carefully. At the same time, the Ethics Committee of the hospital (General Hospital of Western Theater Command) approved the clinical study.

### Sample collection and processing

After venous blood collection, the tube containing whole blood was left to stand for 30 min and centrifuged for 10 min at 4 °C and 3000 rpm; take the upper serum, put each sample into a 1.5-mL EP tube, put 300 µL serum, put it into a −80 °C refrigerator after marking, and wait for GC/LC–MS detection.

Samples stored at −80 °C were thawed at room temperature. Eighty microliters of sample was added to a 1.5-mL Eppendorf tube with 10 μL of 2-chloro-l-phenylalanine (0.3 mg/mL) dissolved in methanol as internal standard, and the tube was vortexed for 10 s. Subsequently, 240 μL of ice-cold mixture of methanol and acetonitrile (2/1, v/v) was added, and the mixtures were vortexed for 1 min, ultrasonicated at ambient temperature (25–28 °C) for 5 min, stored at −20 °C for 10 min. The extract was centrifuged at 12,000 rpm, 4 °C for 10 min. The samples were centrifuged at 12,000 rpm for 10 min at 4 °C. An aliquot of the 150 μL supernatant was transferred to a glass sampling vial for vacuum-dry at room temperature. And 80 μL of 15 mg/mL methoxylamine hydrochloride in pyridine was subsequently added. The resultant mixture was vortexed vigorously for 2 min and incubated at 37 °C for 90 min. Eighty microliters of BSTFA (with 1% TMCS) and 20 μL *n*-hexane were added into the mixture, which was vortexed vigorously for 2 min and then derivatized at 70 °C for 60 min. The samples were placed at ambient temperature for 30 min before GC–MS analysis.

Other 100 μL of sample was added to a 1.5-mL Eppendorf tube with 10 μL of 2-chloro l-phenylalanine (0.3 mg/mL) dissolved in methanol as internal standard, and the tube was vortexed for 10 s. Subsequently, 300 μL of ice-cold mixture of methanol and acetonitrile (2/1, v/v) was added, and the mixtures were vortexed for 1 min, ultrasonicated at ambient temperature (25–28 °C) for 10 min, stored at −20 °C for 30 min. The extract was centrifuged at 13,000 rpm, 4 °C for 15 min. Three hundred milliliters of supernatant in a brown and glass vial was dried in a freeze concentration centrifugal dryer, 0.400 μL mixture of methanol and water (1/4, vol/vol) was added to each sample, samples vortexed for 30 s, then placed at 4 °C for 2 min. Samples were centrifuged at 13,000 rpm, 4 °C for 5 min. The supernatants (150 μL) from each tube were collected using crystal syringes, filtered through 0.22-μm microfilters, and transferred to LC vials. The vials were stored at −80 °C until LC–MS analysis.

### Metabolite measurement

The instrument used for GC analysis this time is Agilent 7890B gas chromatography system and Agilent 5977B MSD system (Agilent Technologies Inc., CA, USA). The instrument used for LC analysis is Vion IMS QTof Mass Spectrometer (Waters Corporation, Milford, USA) and ACQUITY UPLC I-Class system (Waters Corporation, Milford, USA). In order to evaluate the repeatability of the data, QCs were injected every 10 samples throughout the analysis. QC samples were prepared by mixing aliquots of the all samples to be a pooled sample.

The acquired LC–MS raw data were analyzed by the Progenesis QI software (Waters Corporation, Milford, USA) using the following parameters. Precursor tolerance was set 5 ppm, fragment tolerance was set 10 ppm, and retention time (RT) tolerance was set 0.02 min. Internal standard detection parameters were deselected for peak RT alignment, isotopic peaks were excluded for analysis, and noise elimination level was set at 10.00, minimum intensity was set to 15% of base peak intensity. The Excel file was obtained with three-dimensional data sets including m/z, peak RT and peak intensities, and RT–m/z pairs were used as the identifier for each ion. The resulting matrix was further reduced by removing any peaks with missing value (ion intensity = 0) in more than 50% samples. The internal standard was used for data QC (reproducibility).

AnalysisBaseFileConverter software was used to convert the GC–MS raw data (D format) to abf format, and then the abf data were imported into the MD-DIAL software for data processing. Metabolites were annotated through LUG database (untarget database of GC–MS from Lumingbio). After alignment with Statistic Compare component, the raw data array’(.txt) was obtained from raw data with three-dimensional data sets including sample information, peak names (or retention time and m/z) and peak intensities. In the data array, all internal standards and any known pseudopositive peaks (caused by background noise, column bleed, or BSTFA derivatization procedure) were removed. After relative standard deviation of the interior label > 0.3 deleted, all peak strength (peak area) is processed by normalization of multi-interior label according to retention time partition period.

### Multivariate data analysis

Metabolites were identified by R software, based on public databases. Principle component analysis (PCA) and (orthogonal) partial least-squares-discriminant analysis ((O)PLS-DA) were performed to visualize the metabolic difference among experimental groups, after mean centering and unit variance scaling. The Hotelling’s T2 region, shown as an ellipse in score plots of the models, defines the 95% confidence interval of the modeled variation. Variable importance in the projection (VIP) ranks the overall contribution of each variable to the OPLS-DA model, and those variables with VIP > 1 are considered relevant for group discrimination. In this study, the default 7-round cross-validation and 200 response permutation testing were used to guard against overfitting.

### Find key biomarkers and analysis metabolic pathway

The differential metabolites were selected on the basis of the combination of a statistically significant threshold of VIP values obtained from the OPLS-DA model and *p* values from a two-tailed Student’s *t* test on the normalized peak areas from different groups, where metabolites with VIP values > 1.0 and *p* values < 0.05 were considered as differential metabolites. At the same time, we also drew the volcanic map and hierarchical clustering of related metabolites. The volcanic map can be used to visualize the *p* value and fold change (FC) value, which is conducive to screening differential metabolites. FC is the ratio of the average content of the differential metabolite in the two groups. Then, based on the Kyoto Encyclopedia of Genes and Genomes (KEGG), we searched for the metabolic pathways related to these key metabolites, consulted relevant literature, verified their pathological relationship, and finally obtained their metabolic pathway map. It is helpful to understand the mechanism of metabolic pathway changes in different samples by mapping different metabolites.

### Statistical methods

Means ± SDs. The Kolmogorov–Smirnov test was used to check the normality and homogeneity of variance of all data. Student’s 2-sided *t* test was used to compare the results of the two groups, and a one-factor analysis of variance was used to explain more than differences among three groups. The Statistical Package for the Social Sciences, version 25.0 (SPSS, Chicago, IL), was used for statistical analysis.

## Results

### Participants

In this study, 50 normal people, 50 patients with HUA, and 50 patients with AGA were finally included in the study. The basic characteristics and clinical variables are shown in Table 1. All the participants in our study were men. The age of three groups was basically matched, and the normal distribution was obtained through statistical data analysis. The uric acid levels in HUA and AGA groups were high. Among them, AGA groups had low uric acid. Both groups had normal renal function.

**Table 1:** Participant characteristics at the time of sampling

Characteristics	HCG	HUA group	AGA group	p^a^
Number of participants (*n*)	50	50	50	–
Age (y, mean ± SD)	38.42 ± 10.41	36.94 ± 15.22	38.61 ± 12.6	0.075
Uric acid (μmol/L, mean ± SD)	302.8 ± 30.08	526.64 ± 78.73	553.81 ± 152.37	0.003
Creatinine Clearance (ml/min, mean ± SD)	94.96 ± 3.28	92.21 ± 2.34	91.38 ± 2.15	0.582
Specific gravity (mean ± SD)	1.02 ± 0.003	1.00 ± 0.004	1.00 ± 0.00	0.062
Urine pH (mean ± SD)	6.04 ± 0.83	5.59 ± 0.89	5.57 ± 0.59	0.011

HCG: the healthy control group; HUA: asymptomatic hyperuricemia; AGA: acute gouty arthritis; SD: standard deviation

^a^Calculated by one-factor analysis of variance for categorical variables among three group

### Multivariate data analysis base on MS data

**Fig. 1:**
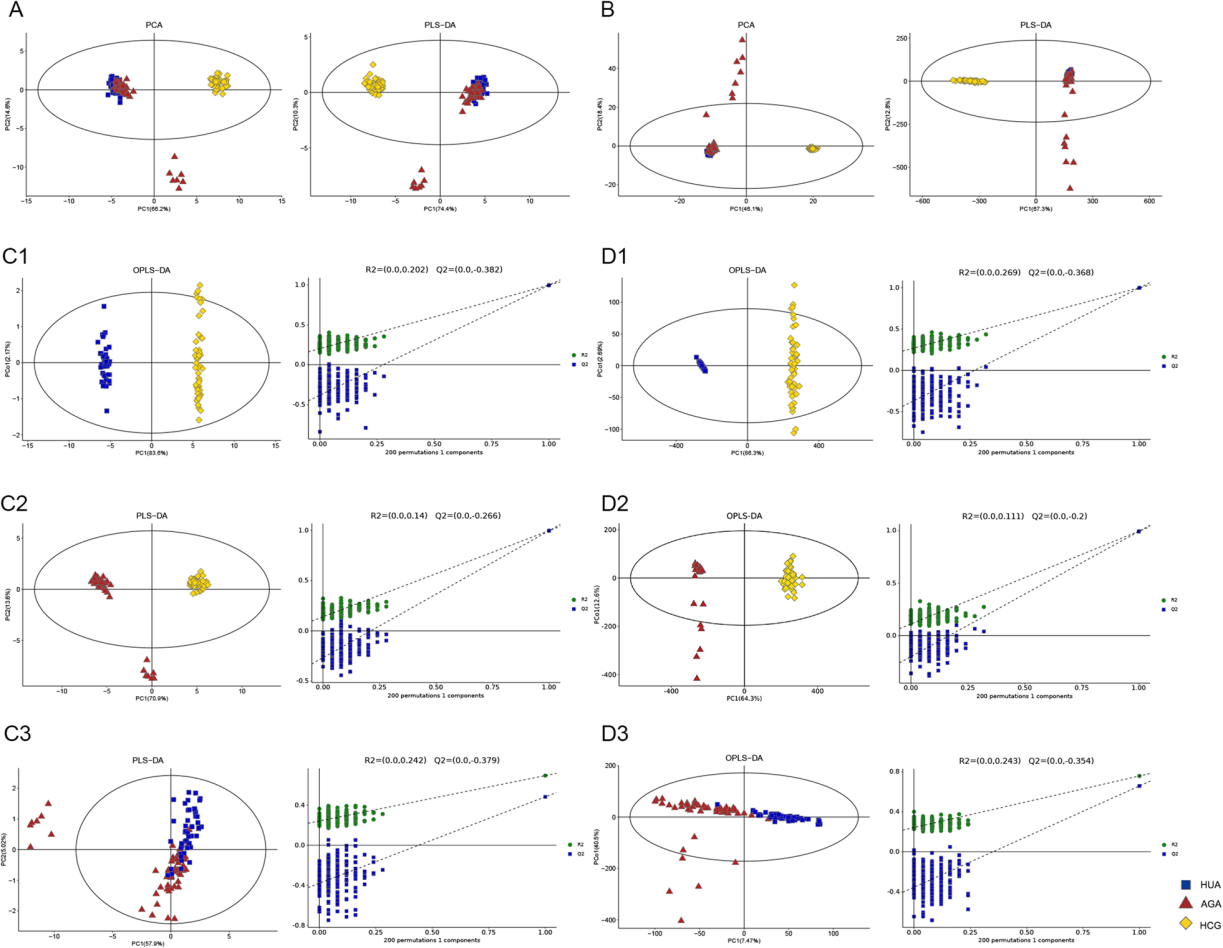
Multivariate date analysis of date between the three groups. **A** PCA score plots and PLS-DA score plots based on the GC–MS. **B** PCA score plots and PLS-DA score plots based on the LC–MS. **C** OPLS-DA score plots and OPLS-DA score plots (left panel) and statistical validation of the corresponding OPLS-DA model by permutation analysis (right panel) based on the GC–MS. **D** OPLS-DA score plots and OPLS-DA score plots (left panel) and statistical validation of the corresponding OPLS-DA model by permutation analysis (right panel) based on the LC–MS. The two coordinate points are relatively far away on the score map, indicating that there is a significant difference between the two samples, and vice versa. The elliptical region represents a 95% confidence interval. HCG: the healthy control group; HUA: asymptomatic hyperuricemia; AGA: acute gouty arthritis

According to the PCA, PLS-DA, and OPLS-DA score chart (Fig. 1), the samples of HUA/AGA and the HCG have obvious separation degree. It can be seen that there is a significant difference in metabolites between HUA/AGA and HCG, and there is also a certain difference between HUA and AGA group. The response ranking test results of OPLS-DA model show that there is no overfitting of the model in this experiment.

**Fig. 2:**
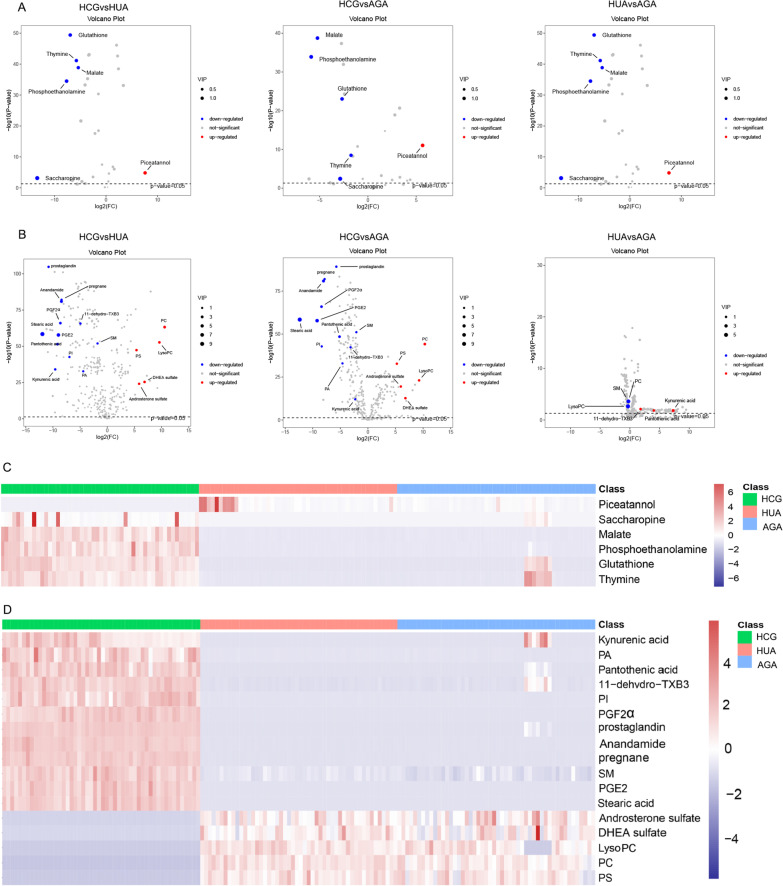
Volcano plot and hierarchical clustering of three groups. **A** Volcano plot based on GC–MS. **B** Volcano plot based on LC–MS. **C** Hierarchical clustering based on GC–MS. **D** Hierarchical clustering based on LC–MS. In (**A**, **B**), the blue dot represents metabolite with a downward trend, red represents metabolites with an upward trend, and the gray origin represents that the change of metabolites is not obvious. The area size of the point is related to the VIP value. In (**C**, **D**), the color from blue to red illustrates that metabolites’ expression abundance is low to high in hierarchical clustering. HCG: the healthy control group; HUA: asymptomatic hyperuricemia; AGA: acute gouty arthritis; LysoPC: lysophosphatidylcholine; PA: phosphatidic acid; PC: phosphatidylcholine; PGE2: Dinoprostone; PGF2α: Dinoprost; PI: phosphatidylinositol; PS: phosphatidylserine; SM: sphingomyelin

Through the volcano map (Fig. 2A, B), *p* value and FC value can be expressed more intuitively. Hierarchical clustering of the expression of all metabolites with significant differences can more directly reflect the relationship between samples and the difference of metabolite expression between different samples (Fig. 2C, D).

### Potential biomarkers and pathway analysis

**Fig. 3:**
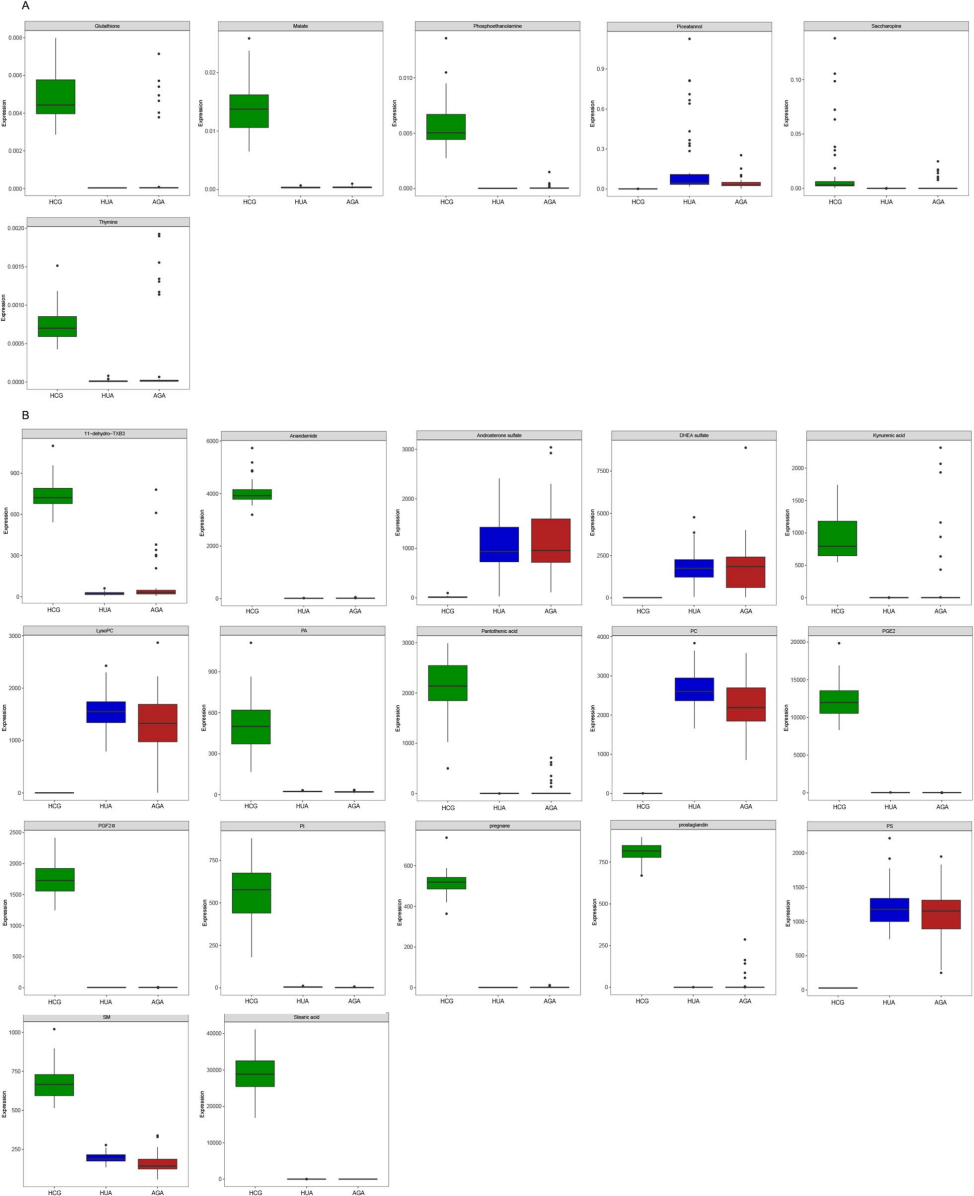
Box-and-whisker plots of selected potential biomarkers. **A** selected potential biomarkers were found by GC–MS. **B** selected potential biomarkers were found by LC–MS. The green box on the left represents the HCG, and the blue box on the middle represents the HUA, the red box on the right represents the AGA. Horizontal line in the middle portion of the box, median; bottom and top boundaries of boxes, lower and upper quartiles; whiskers, 5th and 95th percentiles. HCG: the healthy control group; HUA: asymptomatic hyperuricemia; AGA: acute gouty arthritis; LysoPC: lysophosphatidylcholine; PA: phosphatidic acid; PC: phosphatidylcholine; PGE2: Dinoprostone; PGF2α: Dinoprost; PI: phosphatidylinositol; PS: phosphatidylserine; SM: sphingomyelin

**Fig. 4:**
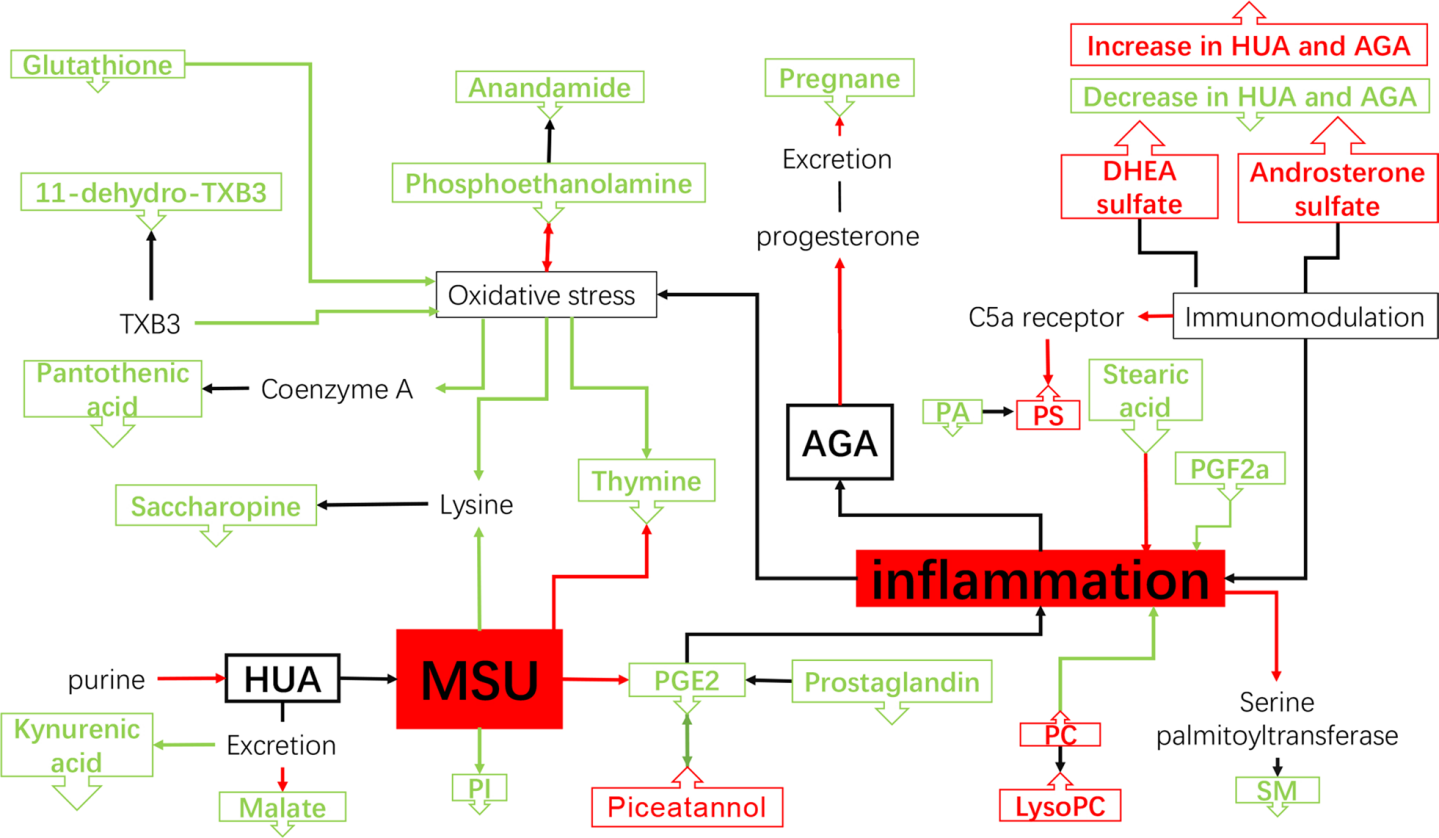
Altered metabolic pathways for the potential biomarkers. The metabolites with red border were upregulated in the HUA/AGA groups, whereas those with green border indicate metabolites that were downregulated. The red arrow represents promotion, the green arrow represents inhibition, and the black arrow represents upstream downstream relationship. HUA: asymptomatic hyperuricemia; AGA: acute gouty arthritis; LysoPC: lysophosphatidylcholine; PA: phosphatidic acid; PC: phosphatidylcholine; PGE2: Dinoprostone; PGF2α: Dinoprost; PI: phosphatidylinositol; PS: phosphatidylserine; SM: sphingomyelin

After multivariate analysis, according to the VIP, FC, and *p* values of metabolites, 23 metabolites were considered as potential biomarkers of HUA and AGA (Table 2). Table 2 shows the specific metabolites specified by GC/LC–MS. Significant differences between groups can also be shown by box-and-whisker plots (Fig. 3). Through database search (KEGG) and literature review, we found that these metabolites are mainly related to uric acid metabolism and oxidative stress. As shown in Fig. 4, we can more intuitively reflect the relationship between these metabolites by drawing the metabolic pathway map of metabolic markers with significant differences.

**Table 2:** Summary of potential biomarkers of three group by serum GC/LC–MS analysis

Metabolites	Changes in HUA (vs HCG)			Changes in AGA (vs HCG)			Changes in AGA (vs HUA)			Data origin
	Status^†^	VIP value^‡^	*p*^§^	Status^†^	VIP value^‡^	*p*^§^	Status^†^	VIP value^‡^	*p*^§^	
Glutathione	↓	1.21	< 0.001	↓	1.16	< 0.001	↓	1.09	< 0.001	GC–MS
Malate	↓	1.06	< 0.001	↓	1.18	< 0.001				GC–MS
Phosphoethanolamine	↓	1.28	< 0.001	↓	1.33	< 0.001	↓	1.35	0.029	GC–MS
Piceatannol	↑	1.14	< 0.001	↑	1.19	< 0.001	↑	1.10	0.001	GC–MS
Saccharopine	↓	1.80	< 0.001	↓	1.60	< 0.001	↓	2.15	0.010	GC–MS
Thymine	↓	1.11	< 0.001	↓	1.01	< 0.001	↓	1.62	< 0.001	GC–MS
11-Dehydro-TXB3	↓	1.50	< 0.001	↓	1.43	< 0.001	↑	1.26	< 0.001	LC–MS
Anandamide	↓	3.61	< 0.001	↓	3.70	< 0.001	–			LC–MS
Androsterone sulfate	↑	1.68	< 0.001	↑	1.72	< 0.001	–			LC–MS
DHEA sulfate	↑	2.23	< 0.001	↑	1.98	< 0.001	–			LC–MS
Kynurenic acid	↓	1.64	< 0.001	↓	1.27	< 0.001	↑	2.17	0.015	LC–MS
LysoPC	↑	2.22	< 0.001	↑	1.87	< 0.001	↓	5.50	0.002	LC–MS
PA	↓	1.18	< 0.001	↓	1.22	< 0.001				LC–MS
Pantothenic acid	↓	2.59	< 0.001	↓	2.61	< 0.001	↑	1.10	0.014	LC–MS
PC	↑	2.91	< 0.001	↑	2.68	< 0.001	↓	4.6.3	< 0.001	LC–MS
PGE2	↓	6.19	< 0.001	↓	6.35	< 0.001	–			LC–MS
PGF2α	↓	2.36	< 0.001	↓	2.43	< 0.001	–			LC–MS
PI	↓	1.28	< 0.001	↓	1.31	< 0.001	–			LC–MS
Pregnane	↓	1.29	< 0.001	↓	1.32	< 0.001	–			LC–MS
Prostaglandin	↓	1.62	< 0.001	↓	1.64	< 0.001	–			LC–MS
PS	↑	1.90	< 0.001	↑	1.80	< 0.001	–			LC–MS
SM	↓	1.22	< 0.001	↓	1.30	< 0.001	↓	1.01	< 0.001	LC–MS
Stearic acid	↓	9.56	< 0.001	↓	9.81	< 0.001	–			LC–MS

HUA: asymptomatic hyperuricemia; AGA: acute gouty arthritis; LysoPC: lysophosphatidylcholine; PA: phosphatidic acid; PC: phosphatidylcholine; PGE2: Dinoprostone; PGF2α: Dinoprost; PI: phosphatidylinositol; PS: phosphatidylserine; SM: sphingomyelin

^†^Relative concentrations: ↑ = upregulated, ↓ = downregulated

^‡^Correlation coefficient and VIP value were obtained from OPLS-DA analysis

^§^*p* value determined from Student’s *t* test

## Discussion

Metabonomics is closely related to the biochemical functions of cells or organisms, so metabonomics is the most important method to understand disease symptoms and biological functions. In this study, the serum metabolites of patients with HUA, AGA, and HCG were analyzed by GC/LC–MS, which comprehensively reflected the changes of specific metabolites in different states of the body. This research method considers multiple metabolite markers as a whole and provides a potential method to explore the progression from normal to HUA and then to AGA, so as to predict the risk of HUA and AGA by using metabolic markers and make early and accurate diagnosis.

This is the first metabonomic study using GC/LC–MS to systematically study the serum of patients with HUA, AGA, and HCG. Through multivariable analysis, we found that there were a large number of metabolites with significant differences among the three groups of serum metabolites, and further studied the metabolic pathways of these metabolites. We found 23 metabolites that may have a good predictive effect on HUA and AGA. Through our study on these metabolites, we found that these metabolites are mainly related to uric acid metabolism and inflammatory response caused by HUA/AGA.

The main pathogenesis of HUA is the disorder of purine metabolism and the decrease of uric acid excretion, which eventually leads to the increase of uric acid concentration in the blood. Through relevant studies, it was found that gout patients with reduced uric acid clearance and normal creatinine clearance showed that the average 24-h urinary excretion of malate increased [9]; we also found that there were lower levels of malate in the serum of HUA and AGA group. Uric acid can inhibit breast cancer resistance protein activity to reduce the excretion of kynurenic acid, resulting in the increase of plasma kynurenic acid level in vivo [10]; however, in the study, we found completely opposite results, which requires further research.

Another study found that the pituitary gonadal system in male gout patients is unbalanced, which is manifested by excessive secretion of progesterone and inhibition of the production of testosterone and estradiol [11]; progesterone can greatly improve the excretion capacity of pregnane [12], which can explain the fact that we found that the concentration of pregnane also decreased in the serum of the HUA and AGA.

When the body's high level of urate reaches a certain threshold, it will induce the body's inflammatory response and trigger AGA [13]. Glutathione can reduce the damage of oxidative stress to the body. In case of HUA and AGA, glutathione in the body is consumed in response to the inflammatory damage of urate [14], resulting in a decrease in its concentration. As an anti-inflammatory substance, TXB3 has a similar effect with glutathione. TXB2 will eventually be metabolized into 11-dehydro-TXB3 in vivo [15]. We found that its concentration also decreased in the HUA and AGA groups. Dinoprostone (PGE2) plays a central role in the inflammatory regulation of AGA (severe pain, redness, and swelling). Piceatannol can prevent the synthesis of PGE2 stimulated by MSU crystal, and PGE2 can also inhibit the production of piceatannol [16]. Stearic acid has also been shown in studies to induce inflammation [17]. But our research has just the opposite result; in our study, we found that the piceatannol concentration in the HUA and AGA group was higher than that in HCG, while the concentration levels of prostaglandin (the precursor of PGE2), stearic acid and PGE2, and Dinoprost (PGF2α) with the same pro-inflammatory effect were decreased. These metabolites may be specific markers of HUA and AGA, which need further study. When urate undergoes oxidation reaction in vivo, it mainly reacts with lysine [18]. The main metabolite of lysine is saccharopine [19]. Saccharopine is significantly lower in the HUA/AGA than HCG, which may be the reason for the oxidation–reduction reaction of a large amount of lysine by urate. At the same time, this inflammatory reaction will promote each other with phosphoethanolamine (PE) resulting in the increase of PE [20], and anandamide is the metabolite of PE [21], but our study also found different results. Androsterone sulfate and dehydroepiandrosterone (DHEA) sulfate may be related to the development of inflammation and immunoregulatory activity [22], but our study shows the opposite trend to other inflammatory diseases. Some studies have found that when the body is in the state of oxidative stress, the body will inhibit the synthesis of coenzyme A in cells [23]. Pantothenic acid is the precursor of coenzyme a synthesis, which can explain the reason for the decrease of serum concentration of pantothenic acid in HUA and AGA. Inflammation can stimulate the release of phosphatidylserine (PS) from complement component 5a receptor 1 to promote the expression of neutrophils [24]; we also found that the concentration of PS in the serum of hyperuricemia group and gout group was higher than that of the normal group. In the inflammatory reaction of the body, phosphatidylcholine (PC) can bind to the platelet-activating factor receptor to achieve the anti-inflammatory effect [25]. Lysophosphatidylcholine (LysoPC) is the hydrolysate of PC [26]; we also found that the concentration levels of lysoPC and PC in HUA/AGA had an increasing trend. During inflammation, the body also stimulates the synthesis of sphingomyelin (SM) by increasing the mRNA expression and activity of serine palmitoyltransferase [27], but the concentration of SM in the serum of HUA and AGA tended to decrease; this also suggests that SM may have diagnostic significance in this pathological process. Some studies have found that uric acid protection of nuclei from ozone-induced degradation can protect thymine [28], and uric acid salt can also cause the concentration of phosphatidylinositol (PI) to increase [29], but our results are just the opposite. Phosphatidic acid (PA) also has a downward trend, and its mechanism is unknown. However, in another study, we have the same results [26].

In addition, through analysis, we found that most of the metabolites also have significant differences between HUA and AGA, and their concentration trends in serum are opposite to those between HUA/AGA and HCG. Some studies have shown that the serum uric acid concentration in AGA may be lower than that in HUA, which is related to the increase of urinary acid excretion [30], which may also be the reason for this result in our study.

In this study, we found three different results from previous gout studies, namely kynurenic acid, thymine, and PI. In addition, we also found 9 metabolites with different trends from conventional inflammation, which are very likely to become specific markers of high uric acid and gout, and deserve further study. With the continuous innovation of metabonomics technology and the improvement of people's requirements for quality of life, scholars pay more attention to the early diagnosis of various diseases. As a new screening technique, metabonomics has been used in the diagnosis of diseases and clinical application. In general, these potential biomarkers found in our study have important biological significance for the diagnosis of HUA and AGA.

## Conclusions

We analyzed 150 serum samples from HUA, AGA, and healthy control group by GC/LC–MS, which proved that GC/LC–MS is a valuable detection method in metabonomics. Through the screening of metabolites and the enrichment analysis of metabolic pathway map, we compared the three groups and found that there were 23 different metabolites, which were mainly related to uric acid metabolism and inflammatory response caused by HUA/AGA. These metabolic changes may bring new hope for early prediction and diagnosis of HUA and AGA.

## Data Availability

The raw data supporting the conclusions of this article will be made available by the authors, without undue reservation.

## Abbreviations

AGA: Acute gouty arthritis
CGA: Chronic gouty arthritis
DHEA: Dehydroepiandrosterone
FC: Fold change
GC: Gas chromatography
HCG: Healthy control group
HUA: Asymptomatic hyperuricemia
KEGG: The Kyoto Encyclopedia of Genes and Genomes
LC: Liquid chromatography
LysoPC: Lysophosphatidylcholine
MSU: Monosodium urate
MS: Mass spectrometry
(O)PLS-DA: (Orthogonal) partial least-squares-discriminant analysis
PA: Phosphatidic acid
PC: Phosphatidylcholine
PCA: Principle component analysis
PE: Phosphoethanolamine
PGE2: Dinoprostone
PGF2α: Dinoprost
PI: Phosphatidylinositol
PS: Phosphatidylserine
RT: Retention time
SM: Sphingomyelin
VIP: Variable importance in the projection
