# Correction: A metabonomic study to explore potential markers of asymptomatic hyperuricemia and acute gouty arthritis

**DOI:** 10.1186/s13018-026-06783-7

**Published:** 2026-03-10

**Authors:** Wei Wang, Jun Kou, Mingmei Zhang, Tao Wang, Wei Li, Yamen Wang, Qingyun Xie, Meng Wei

**Affiliations:** 1 Department of Orthopedics, General Hospital of Western Theater Command, Rongdu Avenue No. 270, Chengdu, 610000 People’s Republic of China; 2 College of Medicine, Southwest Jiaotong University, North Section 1 No. 111, Second Ring Road, Chengdu, 610000 People’s Republic of China; 3 Department of Rheumatism and Immunology, The General Hospital of Western Theater Command, Tianhui Road 270, Chengdu, 610000 People’s Republic of China

**Correction to: Journal of Orthopaedic Surgery and Research (2023) 18:96** 10.1186/s13018-023-03585-z

In this article [1], the third volcano plot in Figure 2A, which was intended to represent the HUAvsAGA comparison, was inadvertently duplicated from the HCGvsHUA comparison group. The incorrect and corrected versions of figure 2 was shown below.

Incorrect Figure 2:
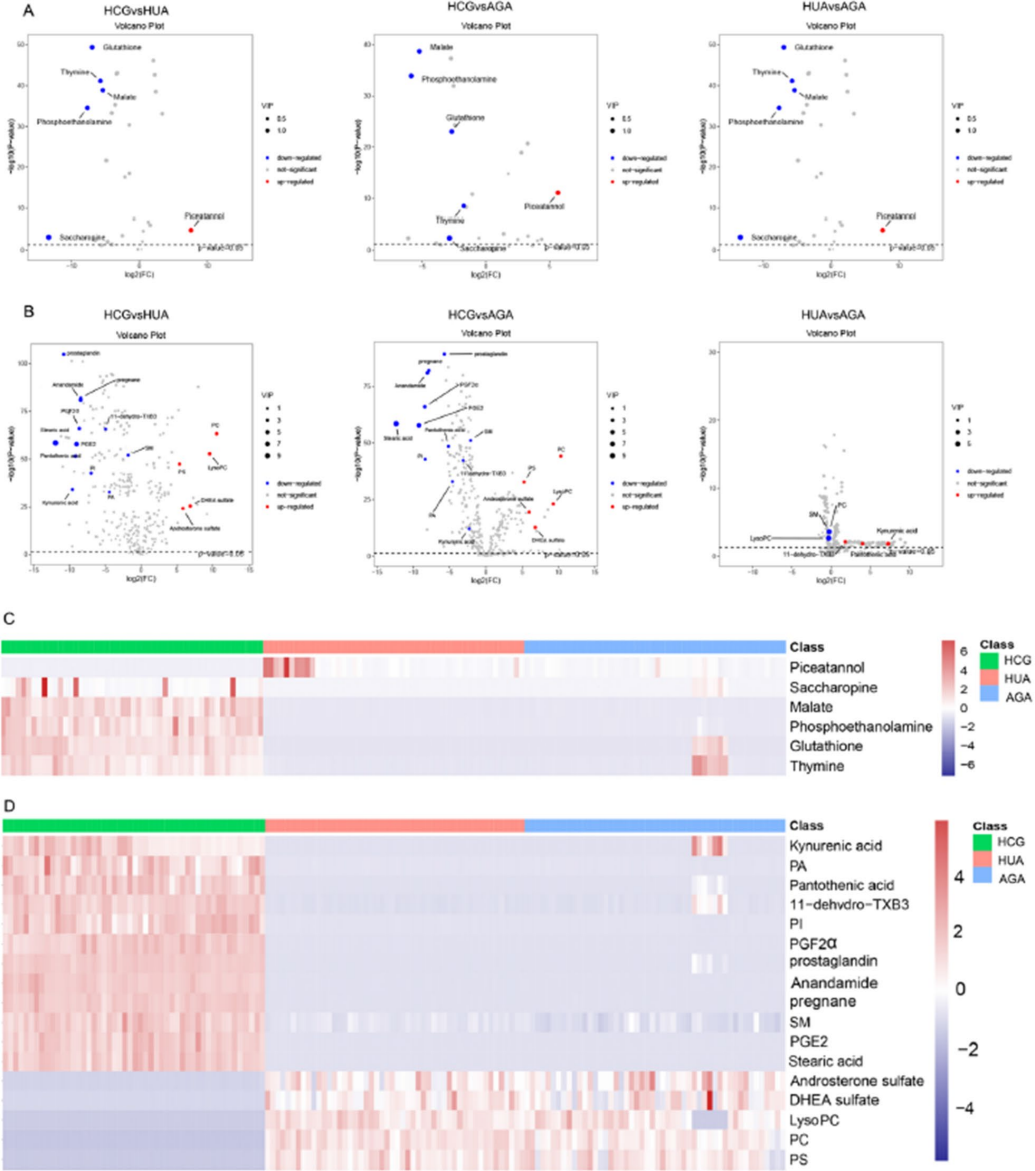


Correct Figure 2:

**Fig. 2:**
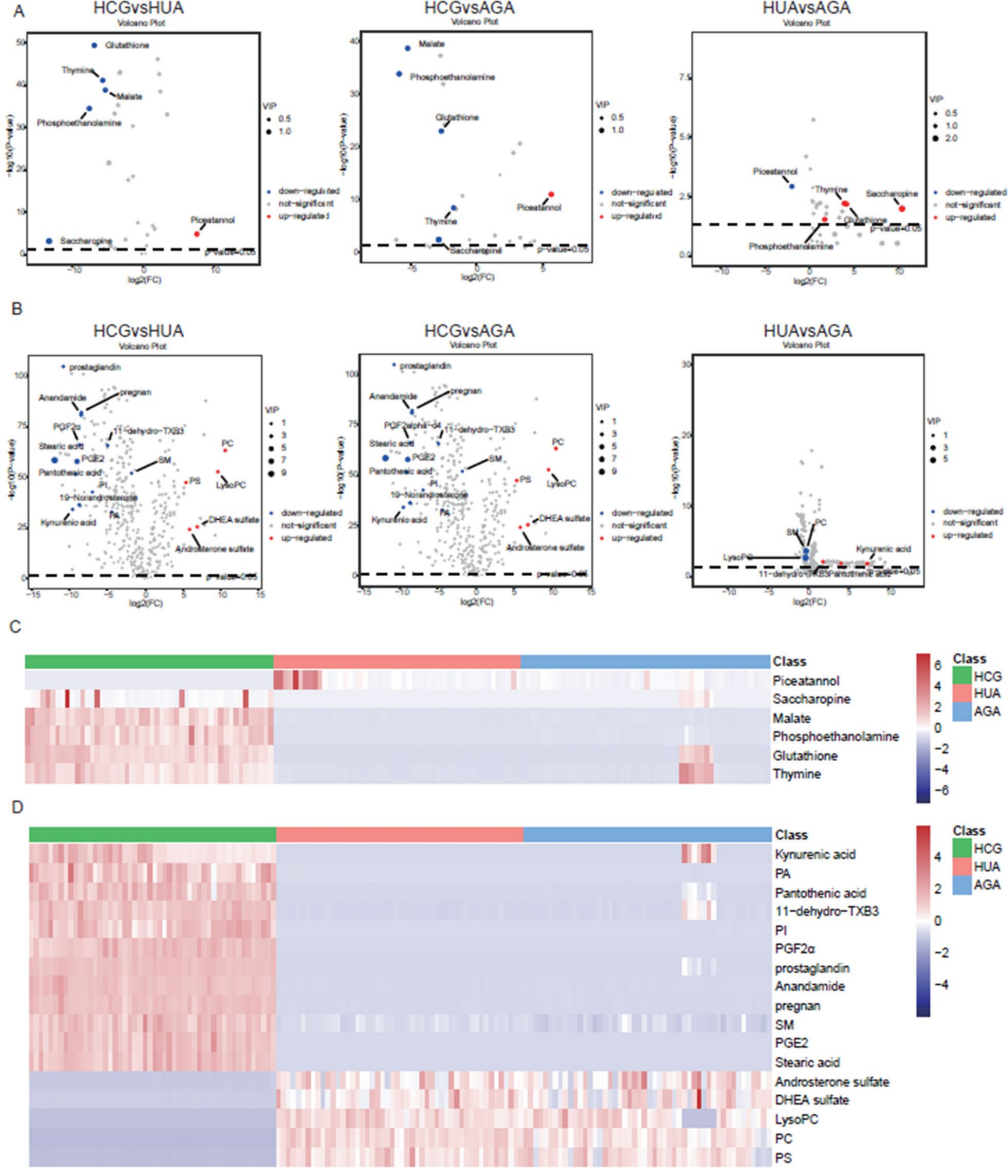
Volcano plot and hierarchical clustering of three groups. **A** Volcano plot based on GC–MS. **B** Volcano plot based on LC–MS. **C** Hierarchical clustering based on GC–MS. **D** Hierarchical clustering based on LC–MS. In (A, B), the blue dot represents metabolite with a downward trend, red represents metabolites with an upward trend, and the gray origin represents that the change of metabolites is not obvious. The area size of the point is related to the VIP value. In (C, D), the color from blue to red illustrates that metabolites’ expression abundance is low to high in hierarchical clustering. HCG: the healthy control group; HUA: asymptomatic hyperuricemia; AGA: acute gouty arthritis; LysoPC: lysophosphatidylcholine; PA: phosphatidic acid; PC: phosphatidylcholine; PGE2: Dinoprostone; PGF2α: Dinoprost; PI: phosphatidylinositol; PS: phosphatidylserine; SM: sphingomyelin
